# Spiking neural networks for nonlinear regression

**DOI:** 10.1098/rsos.231606

**Published:** 2024-05-01

**Authors:** Alexander Henkes, Jason K. Eshraghian, Henning Wessels

**Affiliations:** ^1^ Computational Mechanics Group, ETH Zurich, Zurich, Switzerland; ^2^ Division Data-Driven Modeling of Mechanical Systems, Technical University Braunschweig, Braunschweig, Germany; ^3^ Department of Electrical and Computer Engineering, University of California, Santa Cruz, CA, USA

**Keywords:** artificial neural networks, spiking neural networks, regression, neuromorphic computing

## Abstract

Spiking neural networks (SNN), also often referred to as the third generation of neural networks, carry the potential for a massive reduction in memory and energy consumption over traditional, second-generation neural networks. Inspired by the undisputed efficiency of the human brain, they introduce temporal and neuronal sparsity, which can be exploited by next-generation neuromorphic hardware. Energy efficiency plays a crucial role in many engineering applications, for instance, in structural health monitoring. Machine learning in engineering contexts, especially in data-driven mechanics, focuses on regression. While regression with SNN has already been discussed in a variety of publications, in this contribution, we provide a novel formulation for its accuracy and energy efficiency. In particular, a network topology for decoding binary spike trains to real numbers is introduced, using the membrane potential of spiking neurons. Several different spiking neural architectures, ranging from simple spiking feed-forward to complex spiking long short-term memory neural networks, are derived. Since the proposed architectures do not contain any dense layers, they exploit the full potential of SNN in terms of energy efficiency. At the same time, the accuracy of the proposed SNN architectures is demonstrated by numerical examples, namely different material models. Linear and nonlinear, as well as history-dependent material models, are examined. While this contribution focuses on mechanical examples, the interested reader may regress any custom function by adapting the published source code.

## 1. Introduction

In recent years, artificial neural networks (ANN) have gained much attention in the computational engineering sciences and applied mathematics due to their flexibility and universal approximation capabilities, both for functions [1,2] and operators [3]. Their outstanding but surprising generalizability capabilities are yet to be understood [4]. Their advantages have been used in a variety of applications, including fluid dynamics [5–9], solid mechanics [10–13], micromechanics [14–17], material parameter identification [18–22], constitutive modelling [23–26], fracture mechanics [27,28], microstructure generation [29–31], contact problems [32–35], heat transfer [36–39], uncertainty quantification [40–43], among numerous others. Refer to [44–46] for review publications.

**Figure 1.**
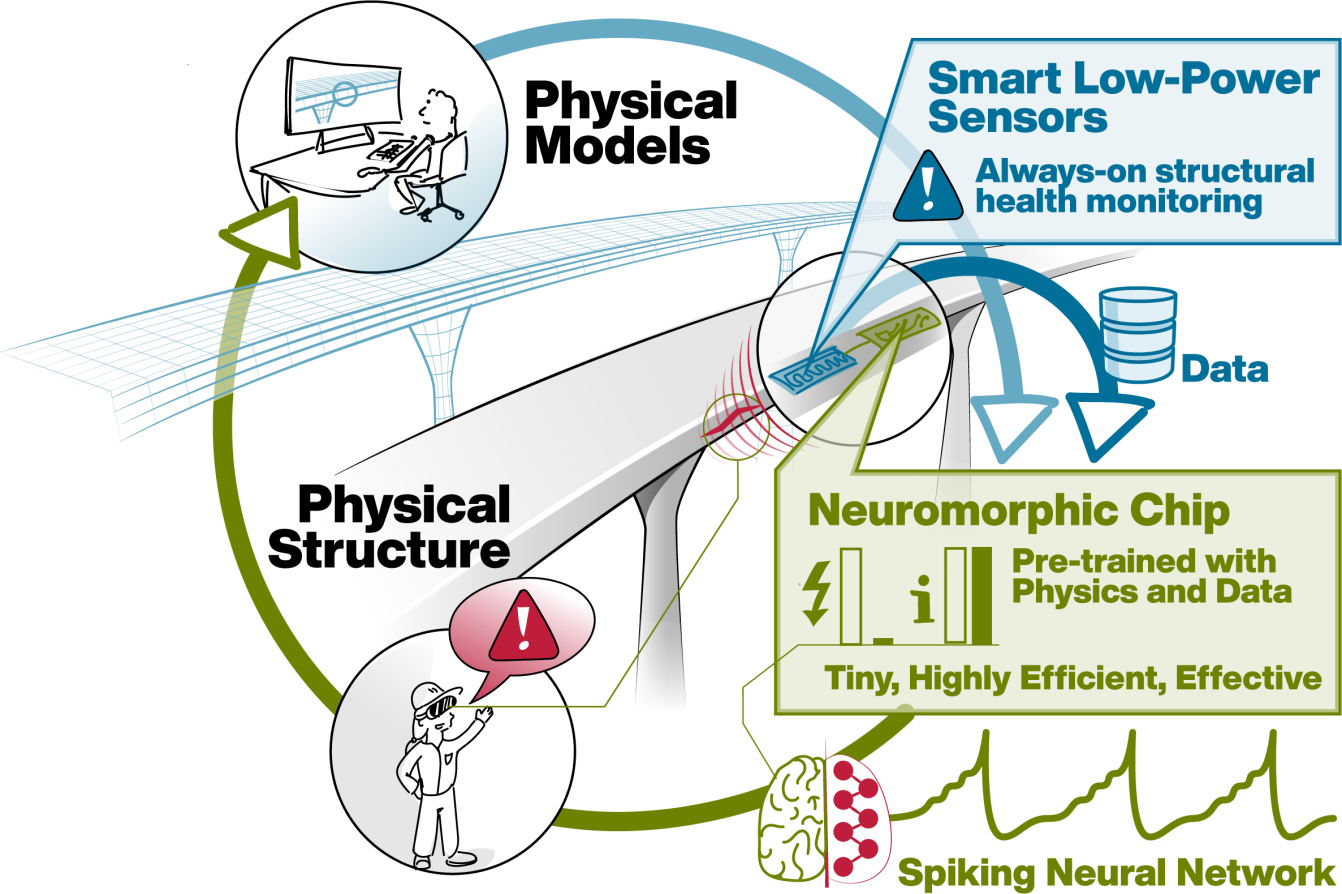
SNN-based regression offers high potential in many energy-critical applications, such as SHM. We expect that, thanks to the advent of data-driven modelling, it will become possible to interpret sensor data directly where it is recorded on neuromorphic hardware as part of embedded devices. Current work is concerned with the solution of partial differential equations on neuromorphic hardware, which will ultimately allow to evaluate and update computational models online.

Despite the success of ANNs, several problems arise alongside their use, such as the need for high-frequency memory access, which leads to high computational power demand [47,48]. This results in huge costs for training and often makes it preferable to run inference in remote servers during deployment. In general, ANNs are most often trained on graphics processing units (GPUs), whose energy consumption is problematic in embedded systems (e.g. sensor devices) as is required in automotive and aerospace applications [49] or structural health monitoring (SHM). Furthermore, high latency during prediction time can arise where acceleration or parallelization is not available. Figure 1 illustrates a potential use case of neuromorphic hardware in SHM. Using concepts from data-driven mechanics, sensor data collected during service life may be interpreted with the aid of high-dimensional (computational) physical models, which so far have only been used to design infrastructures or products. Thanks to active research, the computational efficiency of such advanced monitoring concepts is rapidly increasing (e.g. [50], for a recent contribution). A bottleneck for practical online applications, however, remains the aforementioned hardware constraints, namely, energy demand and latency. Regression using spiking neural networks (SNN) on neuromorphic hardware may contribute to overcome persisting limitations.

Originally motivated by the human brain, today’s traditional ANN architectures are an oversimplification of biology, relying on dense matrix multiplication. From a numerical and computational hardware point of view, dense matrix multiplication is often suboptimal. Sparsity is thought to be favourable as it reduces dependence on memory access and data communication [51]. In contrast, the human brain is much more efficient, where neurons are considered to be sparsely activated [52]. This stems from the fact that the brain uses sparse electronic signals for information transmission instead of dense activations. This leads to remarkable capabilities by using only approximately 10–20 W of energy. One attempt to overcome these drawbacks of ANNs is to introduce the information transmission mechanism of biological neurons into network architectures. These networks are called SNN due to the electronic impulses or spikes used for communication between neurons [53]. This leads to sparse activations, which can be efficiently exploited by neuromorphic hardware, such as Loihi [54], SpiNNaker [55] and TrueNorth [56]. It has been shown that these specialized hardware chips can reduce the energy consumption of neural network-based processes by factors of up to ×1000 [54,57–60]. Apart from their energy efficiency in prediction, recent attempts to increase the training efficiency of SNN can be found in [61–64].

What was classically in the domain of neuroscientists recently has been investigated in the context of deep learning, for example, the adoption of SNNs to supervised learning as popularized with traditional ANNs in frameworks, such as TensorFlow [65] and PyTorch [66], resulting in similar frameworks for spiking deep learning like snnTorch [67]. Some applications of spiking deep learning include image processing using a spiking ResNet [68] and temporal data processing using spiking long short-term memory neural network (SLSTM) variants [69,70]. A combination of spiking convolutional neural networks and LSTMs was proposed in Yang *et al*. [71]. SNNs have been used for image segmentation [72] and localization [73,74].

To the best of the authors’ knowledge, the full potential of energy-efficient regression modelling using SNNs has up to now not yet been fully exploited. In Iannella and Back [75], an architecture using *inter-spike interval temporal encoding* has been proposed, where the functions learned were limited to piecewise constant functions. In Gehrig *et al*. [76], a SNN was used for the regression of angular velocities of a rotating event camera. Building on these results, Rançon *et al.* [77] proposed a SNN for depth reconstruction. Both works rely on fully connected decoders for real-valued output, which increases the energy demand compared with an architecture relying only on SNN predictions. This is due to the increased number of dense matrix multiplications in fully connected layers. In Kahana *et al*. [78], a DeepONet [79] using SNN was proposed, which used a floating point decoding scheme to regress simple one-dimensional functions. This decoding scheme led to non-smooth and staircase-like predictions. In Shrestha and Orchard [80], gradient descent was applied to learn spike times. Here, as in the present contribution, the membrane potential is used, but to tweak the occurrence of the first spikes in the SNN. In Eshraghian *et al*. [81], classification problems using the membrane potential in the context of memristor-based hardware were explored, but no regression tasks. Recently, a SNN has been proposed in a computational mechanics context in Tandale and Stoffel [82], which builds up on our approach developed in the preprint [83]. However, again fully connected layers were added, which counteract the energy efficiency.

The focus of the present work lies on network architectures that come along without any fully connected layers and are therefore optimally suited for neuromorphic hardware, which is specifically designed for SNNs. The proposed regression approach works without fully connected dense encoders/decoders by using only spiking layers. The output of the proposed networks is real-valued, continuous functions and can represent highly nonlinear, history-dependent data accurately.

As regression problems are omnipresent in engineering sciences, a flexible and broadly applicable framework would enable SNNs to be used in a variety of engineering applications and further unfold the potential of neuromorphic hardware. To this end, the present study aims towards the following key contributions:

— *Introduction of spiking neural network*s. Concise introduction of this emerging technique. Open source benchmark code for the research community, fostering further developments of SNN in mechanics and applied mathematics (see also Data accessibility section).
— *History-dependent regression framework*. We demonstrate that SNNs can model systems that exhibit hysteresis, thus history-dependent behaviour. We present a flexible framework to use SNNs in complex regression tasks, demonstrated by means of a nonlinear material model, namely isotropic hardening plasticity.
— *Efficiency, sparsity and latency*. We benchmark our SNN for neuromorphic hardware in terms of memory and energy consumption as compared with non-spiking equivalent networks, demonstrating that our approach is much more efficient with respect to memory and power consumption, making neural networks more sustainable. This is enabled by a membrane potential-based decoding scheme, which does not rely on fully connected decoders. While in our study an emulator is used, deployment on neuromorphic hardware allows highly efficient usage in embedded environments.

The present work intends to introduce this important novel technique to the community of computational mechanics and applied mathematics. To concentrate on the novelties and to keep the presentation concise, we restrict ourselves to one-dimensional regression problems. However, the framework is not restricted to single-variable regression and is easily applicable to a multi-variable regression.

The remainder of this article is structured as follows. In §2, the basic notations of SNNs are derived from traditional ANNs. A simple spiking counterpart to the classical densely connected feed-forward neural network is introduced. After that, our regression SNN topology is proposed. Starting with a linear regression example (linear elasticity), we point out the problems arising in SNN regression. This basic architecture is extended towards recurrent feedback loops in §3. The ability of these recurrent SNNs is showcased on a nonlinear material model. To process history-dependent regression tasks with dependencies over a large number of time steps, a spiking LSTM is introduced in §4. An application to a history-dependent plasticity model shows that SNNs can achieve similar accuracy as their traditional counterparts while being much more efficient. The paper closes with a conclusion and an outlook towards future research directions in §5. For the code accompanying this manuscript, see the Data accessibility section at the end of this manuscript.

## 2. Spiking neural networks for regression

SNNs are considered to be the third generation of neural networks. While the first generation was restricted to shallow networks, the second generation is characterized by deep architectures. A broad use of second-generation neural networks has been enabled by the availability of automatic differentiation and software frameworks such as TensorFlow [65]. To introduce SNN, we compare them with their well-known second-generation counterparts.

### 2.1. Second-generation neural networks

An ANN is a parametrized, nonlinear function composition. The universal function approximation theorem [1] states that arbitrary Borel measurable functions can be approximated with ANNs. There are several different architectures for ANNs, for example, feed-forward, recurrent or convolutional networks, which can be found in standard references such as [84–88]. Following Hauser [89], most ANN formulations can be unified. An ANN N , more precisely, *a densely connected feed-forward neural network*, is a function from an input space $R^{d_{x}}$ to an output space $R^{d_{y}}$ , defined by a composition of nonlinear functions $h^{\left(l\right)}$ , such that


(2.1) $$\begin{array}{rl} N:R^{d_{x}} & \to R^{d_{y}}\\ x & \mapsto N(x)=h^{\left(l\right)}\circ \ldots \circ h^{\left(0\right)}=y,\\ l & =1,\ldots ,n_{L}. \end{array}$$

Here, *x* denotes an *input vector* of dimension $d_{x}$ and *y* an *output vector* of dimension $d_{y}$ . The nonlinear functions $h^{\left(l\right)}$ are called *layers* and define an l -fold composition, mapping input vectors to output vectors. Consequently, the first layer $h^{\left(0\right)}$ is defined as the *input layer* and the last layer $h^{\left(n_{L}\right)}$ as the *output layer*, such that


(2.2) $$h^{\left(0\right)}=x\in R^{d_{x}},\;h^{\left(n_{L}\right)}=y\in R^{d_{y}}.$$

**Figure 2.**
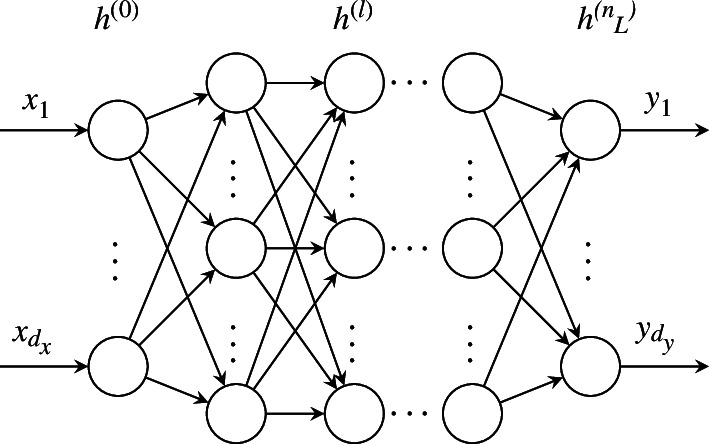
Densely connected feed-forward neural network topology of an ANN N(x;θ) as described in equation (2.1).

The layers $h^{\left(l\right)}$ between the input and output layers, called *hidden layers*, are defined as


(2.3) $$\begin{array}{rl} h^{\left(l\right)} & =\left\{h_{\eta }^{\left(l\right)},\;\eta =1,\ldots ,n_{u}\right\},\\ h_{\eta }^{\left(l\right)} & =\phi^{\left(l\right)}\left(W_{\eta }^{\left(l\right)}h^{\left(l-1\right)}\right), \end{array}$$

where $h_{\eta }^{\left(l\right)}$ is the *n*-th neural unit of the *l*-th layer $h^{\left(l\right)}$ , $n_{u}$ denotes the total number of neural units per layer, $W_{\eta }^{\left(l\right)}$ is the *weight vector of the n*-th neural unit in the *l*-th layer $h^{\left(l\right)}$ and $h^{\left(l-1\right)}$ is the output of the preceding layer, where bias terms are absorbed [86]. Furthermore, $\phi^{\left(l\right)}:R\to R$ is a nonlinear activation function. All weight vectors $W_{\eta }^{\left(l\right)}$ of all layers $h^{\left(l\right)}$ can be gathered in a single expression, such that


(2.4) $$\theta_{\text{ANN}}=\left\{W_{\eta }^{\left(l\right)}\right\},$$

where θ inherits all parameters of the ANN N(x) from equation (2.1). Consequently, the notation N(x;θ) emphasizes the dependency of the outcome of an ANN on the input on one hand and the current realization of the weights on the other hand. The specific combination of layers $h^{\left(l\right)}$ from equation (2.3), neural units $h_{\eta }^{\left(l\right)}$ and activation functions $\phi^{\left(l\right)}$ from equation (2.3) is called the *topology* of the ANN N(x;θ) . The weights *θ* from equation (2.4) are typically found by gradient-based optimization with respect to a task-specific *loss function* [85]. An illustration of a densely connected feed-forward ANN is shown in figure 2.

It can be seen that the ANN described in equation (2.1) takes an input and produces an output, one at a time. If history-dependent input and output data and are considered, the formulation of the hidden layers reads


(2.5) $$h_{t}^{\left(l\right)}=\left\{h_{\eta ,t}^{\left(l\right)},\eta =1,...,n_{u},t=0,...d_{t}\right\},\;h_{\eta ,t}^{\left(l\right)}=\phi^{\left(l\right)}(W_{\eta }^{\left(l\right)}h_{t}^{\left(l-1\right)}),$$

where the time component is discrete. This can be understood as processing each discrete-time slice of the input vector of the preceding layer $h_{t=0}^{\left(l-1\right)}\to h_{t=1}^{\left(l-1\right)}\to ,...,\to h_{t=d_{t}}^{\left(l-1\right)}$ sequentially, where the weights $W_{\eta }^{\left(l\right)}$ are shared over all time steps. At this stage, the formulation in equation (2.5) is purely notational, as there is no connection of the weights through different time steps.

### 2.2. Spiking neural networks

A SNN can be seen as a history-dependent ANN, which introduces memory effects by means of biologically inspired processes. Standard works in theoretical neuroscience include the studies of Dayan and Abbott [90], Izhikevich [91] and Gerstner *et al*. [92]. Several overviews of SNNs with respect to deep learning can be found in [93–95].

**Figure 3.**
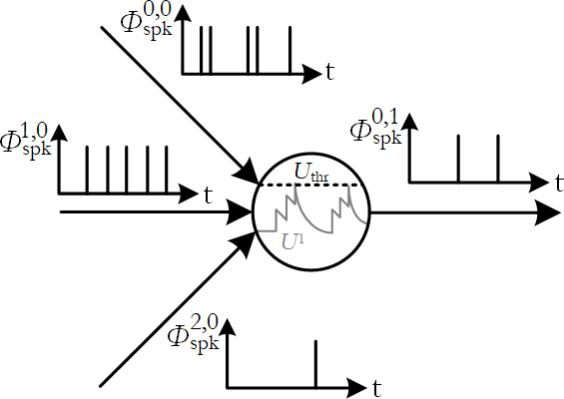
Spiking neuron dynamics. Input spikes $\phi_{spk}^{i,0}$ trigger changes in the membrane potential $U^{1}$ , which when sufficiently excited beyond a threshold $U_{thr}$ causes the neuron to emit an output spike $\phi_{spk}^{j,1}$ .


To this end, the activation function $\phi^{\left(l\right)}$ in equation (2.5) can be formulated as


(2.6) $$\begin{array}{r} \phi_{\text{spk},t}^{\left(l\right)}=\left\{ \begin{array}{l} 1,\;U_{\eta ,t}^{\left(l\right)}\geq U_{\text{thr},\eta }^{\left(l\right)}\\ 0,\;U_{\eta ,t}^{\left(l\right)}<U_{\text{thr},\eta }^{\left(l\right)}, \end{array}\right. \end{array}$$

with


(2.7) $$U_{\eta ,t}^{\left(l\right)}=\beta_{\eta }^{\left(l\right)}U_{\eta ,t-1}^{\left(l\right)}+W_{\eta }^{\left(l\right)}h_{t}^{\left(l-1\right)}-\phi_{\text{spk},t-1}^{\left(l\right)}U_{\text{thr},\eta }^{\left(l\right)},$$

where $U_{\eta ,t}^{\left(l\right)}$ is the membrane potential of the η -th neural unit at time t , $U_{\text{thr},\eta }^{\left(l\right)}$ denotes the membrane threshold, $\beta_{\eta }^{\left(l\right)}$ is the membrane potential decay rate and $W_{\eta }^{\left(l\right)}h_{t}^{\left(l-1\right)}$ is the standard ANN weight multiplied with the preceding layer of the current time step, respectively, see equation (2.5). Basically, the SNN activation restricts the neural unit to output discrete pulses $\left(\phi_{\text{spk}}=1\right)$ if the membrane threshold is reached by the time-evolving membrane potential, or to remains silent $\left(\phi_{\text{spk}}=0\right)$ . These pulses are called *spikes*, see Figure 3. The last summand in equation (2.6), $-\phi_{\text{spk},t-1}^{\left(l\right)}U_{\text{thr},\eta }^{\left(l\right)}$ , is called the *reset mechanism* and resets the membrane potential by the threshold potential once a spike is emitted. The membrane threshold and membrane potential decay rate can be optimized during training, such that the optimization parameters of a SNN are

(2.8) $$\theta_{\text{SNN}}=\left\{W_{\eta }^{\left(l\right)},\beta_{\eta }^{\left(l\right)},U_{\text{thr},\eta }^{\left(l\right)}\right\}.$$

The SNN formulation in equation (2.7) is called the *leaky integrate and fire* (LIF) neuron model, and is one of the most widely used models in spike-based deep learning. It can be seen as the baseline SNN and plays a similar role as densely connected feed-forward ANN in classical deep learning. Equation (2.7) can also be interpreted as the explicit forward Euler solution of an ordinary differential equation, describing the time variation of the membrane potential, see Eshraghian *et al*. [67] for details.

The main difference between SNNs and classical ANNs lies in the way information is processed and propagated through the network from neuron to neuron. In standard ANNs, inputs, hidden layers and output vectors are handled via dense matrices. In SNN, sparsity is introduced by using spikes, which are single events expressed via a Dirac delta function or a discrete pulse in continuous or discrete settings, respectively. A group of spikes over time is called a *spike train* $i=\left[i_{t},\;t=0,...,n_{t}\right]$ . To this end, a spiking neuron is subjected to a spike train over a time interval, consisting of spikes (1) or zero input (0). The membrane potential $U_{\eta ,t}^{\left(l\right)}$ is modulated with incoming spikes $i_{t}$ . In the absence of input spikes, the membrane voltage decays over time due to the membrane decay rate $\beta_{\eta }^{\left(l\right)}$ . The absence of spikes introduces sparsity because, in every time step, the neural unit output is constrained to either 0 or 1. This fact can be exploited on *neuromorphic hardware*, where memory and synaptic weights need only be accessed if a spike is apparent. Otherwise, no information is transmitted. In contrast, conventional ANNs do not leverage sparsely activated neurons, and most deep learning accelerators, such as GPUs or tensor processing units (TPUs), are correspondingly not optimized for it.

### 2.3. Spiking neural networks training

**Figure 4.**
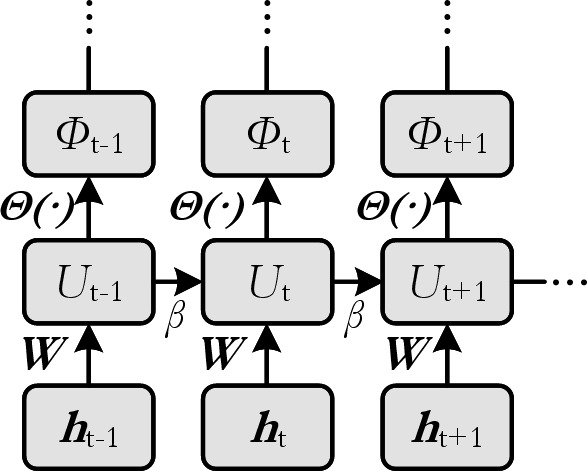
Computational graph of an unrolled SNN. Forward pass.

**Figure 5.**
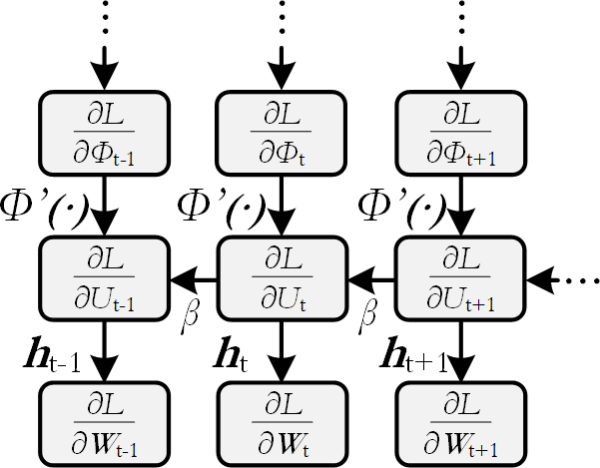
Computational graph of an unrolled SNN. Backward pass.

Unfortunately, the spiking activation $\phi_{\text{spk},t}^{\left(l\right)}$ in equation (2.6) is non-differentiable. To use the backpropagation algorithm from standard ANNs, the activation is replaced using a *surrogate gradient* during the backward pass. Several different formulations have been proposed (e.g. [51,96,97]). In this work, the *arcus tangent surrogate activation* from Fang *et al*. [98] is used,


(2.9) $$\phi_{\text{surr}}\left(x\right)=\frac{1}{\pi }\arctan\left(\pi x\right),\;\phi_{\text{surr}}^{\prime }\left(x\right)=\frac{1}{1+{\left(\pi x\right)}^{2}},$$

for some input x . The surrogate $\phi_{\text{surr}}\left(x\right)$ is continuously differentiable and preserves the gradient dynamics of the network. Thus, for training using backpropagation and its variants, $\phi_{\text{surr}}^{\prime }$ is employed. Illustrations can be found in figures 4 and 5.

### 2.4. Network topology

**Figure 6.**
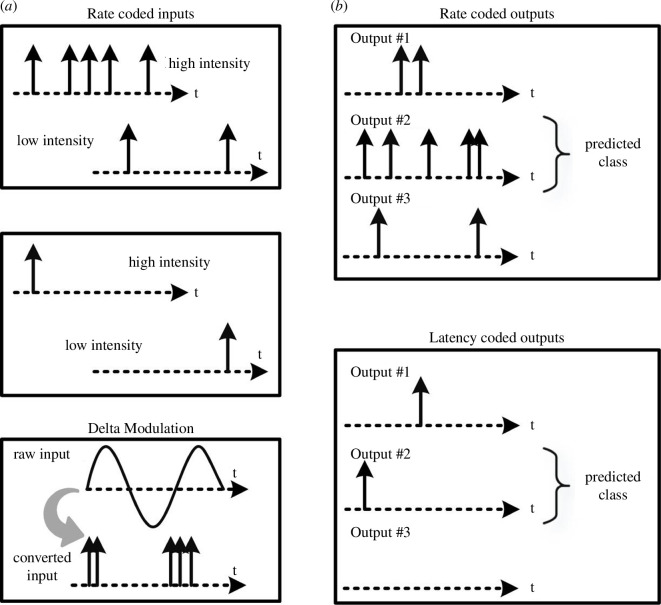
A sample of spike-based encoding and decoding strategies. (*a*): Real-valued inputs are encoded into spikes by means of different strategies [67], for example: (i) high intensities or large values result in a large number of spikes (top left); (ii) high intensities or large values result in early spike firing (centre left); and (iii) delta modulation where spikes are produced for positive gradients of the input function (bottom left). (*b*): In classification, the predicted class is determined via (i) the number of spikes (top right) or (ii) the first occurrence of spikes (bottom right). Regression is introduced in §2.2.

The key question for using SNN in regression is how to transform real input values into binary spikes and binary spike information at the output layer back to real numbers. The former task is called *spike encoding*, whereas the latter is called *spike decoding*. Popular forms of encoding include *rate encoding*, *latency encoding, delta modulation*, among others. Similarly, different decoding strategies exist, such as *rate decoding* and *latency decoding*. An illustration of various encoding and decoding strategies is shown in figure 6. Refer to Eshraghian *et al*. [67] for an overview and detailed description. In this work, a *constant current injection* is chosen for the encoding part, whereas a novel *population voting on membrane potential* approach is chosen for the decoding part. It will be demonstrated throughout the numerical examples that, with the proposed spike decoding scheme, accurate and efficient SNN regression is made possible.

All network topologies used in the upcoming numerical examples follow a general scheme, which is flexible and suited for regression tasks. First, the real input $x_{t}$ is provided as a constant input to the first layer $h_{t}^{\left(0\right)}$ , for all time steps *t*, such that


(2.10) $$h_{t}^{\left(0\right)}\left(x_{t}\right)=h_{t}^{\text{const}}\left(x_{t}\right)=x_{t}\;\forall \;t\in \left[0,d_{t}\right].$$

Then, several SNN layers $h_{t}^{\left(l\right)}$ follow, where the exact formulation will be given for every numerical example. The output of the last spiking layer $h_{t}^{\left(n_{L}\right)}$ is transformed into a *decoding layer* $h_{t}^{\text{dec}}$ , which takes the membrane potential of every time step as input and outputs real numbers


(2.11) $$h_{t}^{\text{dec}}=\beta_{\eta }^{\left(l\right)}U_{\eta ,t-1}^{\left(l\right)}+W_{\eta }^{\left(l\right)}h_{t}^{\left(l-1\right)},$$

which is essentially the formulation of equation (2.7), where no spikes and reset mechanisms are used. The transformed values are then transferred to a ‘population voting layer’, where the output of all neurons of the decoding layer are averaged to give real numbers. This results in


(2.12) $$h_{t}^{pop}=\frac{1}{n_{o}}\sum_{n_{o}}\left(\beta_{n}^{\left(l\right)}U_{\eta ,t-1}^{\left(l\right)}+\;W_{n}^{\left(l\right)}h_{n}^{\left(l-1\right)}\right),$$

where *n*_o_ denotes the number of neurons in the population voting layer and again, no spikes or reset mechanisms are used.

The final spiking regression topology network S can be written as


(2.13) $$\begin{array}{rlr} S:R^{d_{t}\times d_{x}} & \to R^{d_{t}\times d_{y}} & \\ x_{t} & \mapsto h_{t}^{\text{pop}}\circ h_{t}^{\text{dec}}\circ h_{t}^{\left(n_{L}\right)}\circ \ldots \circ h_{t}^{\left(l\right)}\circ \ldots & \\ & \ldots \circ h_{t}^{\left(1\right)}\circ h_{t}^{\text{const}}\left(x_{t}\right)=y_{t}. & \end{array}$$

To summarize, information flows in the following form:

— The input layer $h_{t}^{\text{const}}=x_{t}$ equation (2.10) takes constant current (real numbers $x_{t}$ ).
— The input is then transformed into binary spikes in the spiking layers $h_{t}^{\left(l\right)}$ (equation (2.6))
— and transformed back into real numbers in the translation layer $h_{t}^{\text{dec}}$ Equation (2.11)().
— The output is formed in the population layer $h_{t}^{\text{pop}}$ Equation (2.12).

**Figure 7.**
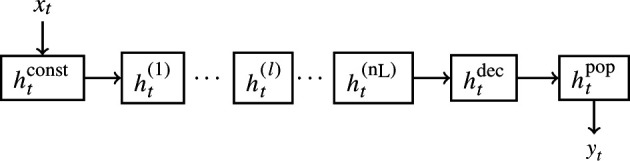
Topology of the spiking regression network is introduced in §2.1.

A graphical interpretation is given in figure 7.

For all the following numerical examples, the AdamW optimizer from Loshchilov and Hutter [99] is used. The parameters are set as follows: learning rate $\alpha =1\times 10^{-3}$ , exponential decay rates for the first- and second-moment estimates $\beta_{1}=0.9$ and $\beta_{2}=0.999$ , respectively, weight decay λ=0.01 . The training was carried out on a Nvidia GeForce RTX 3090 GPU using snnTorch [67] and PyTorch [66]. In this work, the mean relative error E is used, which is defined as


(2.14) $$E_{∙}(∙)=\frac{1}{n_{s}}\sum_{i=1}^{n_{s}}\frac{||{\hat{∙}}_{i}-{∙}_{i}|{|}_{2}}{||{\hat{∙}}_{i}|{|}_{2}},$$

for some input ∙ and baseline $\hat{∙}$ . If the error is reported for all time steps, ∙ is a vector containing the values of all time steps. If the error is reported for the last time step, ∙ is equal to the last component of the corresponding history-dependent vector.

### 2.5. Numerical experiment: linear regression

We first study the performance of the proposed LIF topology in a simple linear regression problem. To this end, the general model described in §2.1 with LIF defined in equation (2.7) is used, resulting in the following network topology.


(2.15) $$S\left(\varepsilon_{t}\right)=h_{t}^{\text{pop}}\circ h_{t}^{\text{dec}}\circ h_{t}^{\text{LIF}}\circ h_{t}^{\text{LIF}}\circ h_{t}^{\text{LIF}}\circ h_{t}^{\text{const}}\left(\varepsilon_{t}\right)=\sigma_{t}.$$

To begin with, a simple linear elastic material model with strains in the range of ε=[0,0.001] and fixed Young’s modulus $E=2.1\times 10^{5}$ MPa is considered, such that the resulting stress σ is


(2.16) σ=Eε.

The training data consists of strain input, uniformly sampled in the interval ε=[0,0.001] , and stress output calculated according to equation (2.16). Three datasets are generated from equation (2.16), namely, training set, validation set and test set consisting each of $n_{\text{train}}=n_{\text{val}}=n_{\text{test}}=1024$ samples. All three datasets are standardized using the mean and standard deviation from the training set. The batch size is chosen as $n_{\text{batch}}=1024$ . The number of neurons $n_{u}$ is chosen as $n_{u}=128$ and is kept constant over all layers. The training is carried out for $2\times 10^{3}$ epochs. The model performing best on the validation set is chosen for subsequent evaluations. The mean relative error accumulated over all time steps and the mean relative error of the last time step with respect to the test set are reported.

**Figure 8.**
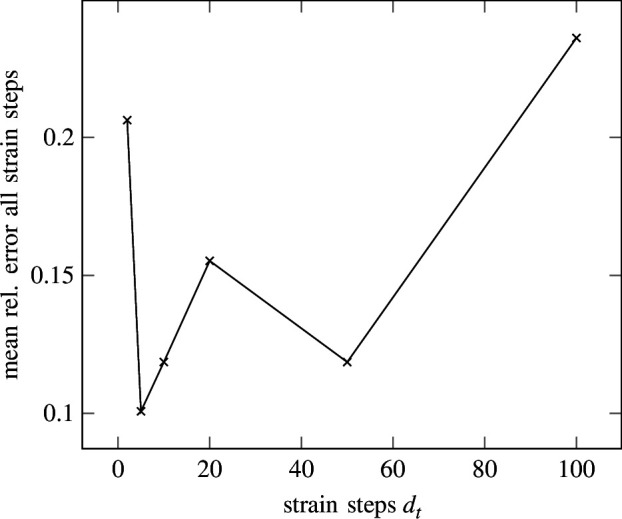
Elasticity—error of all strain (time) steps. Mean relative error for all strain steps with respect to the different total number of strain steps. The error is rising for a larger number of strain steps.

Although the linear regression problem is a time-independent problem, SNN has an inherent time dependency. Instead of time, the SNN is thus trained on a sequence of strain steps Δε . We investigate the effect of the number of strain (time) steps on the prediction accuracy, with results illustrated in figure 8.

**Figure 9.**
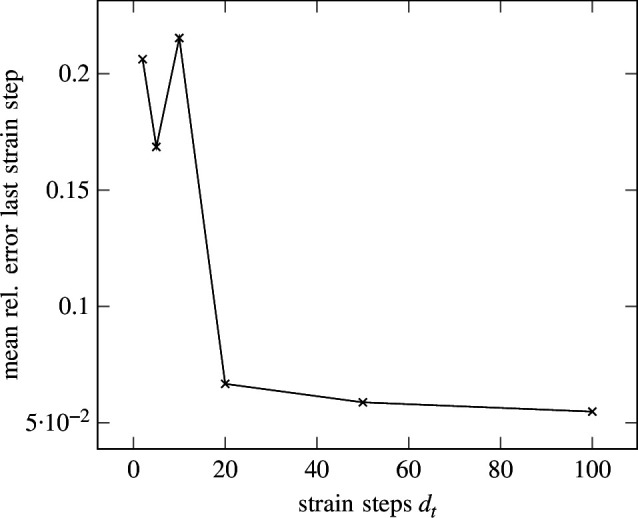
Elasticity—error at last strain (time) step. Mean relative error for the last strain step with respect to the different total number of strain steps. The error is converging for a larger number of strain steps.

**Figure 10.**
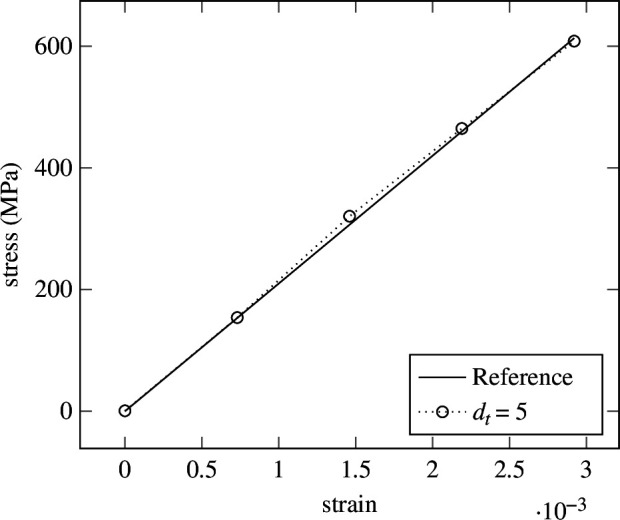
Elasticity—prediction in five strain (time) steps. Prediction of the LIF from equati[Disp-formula uFD2_15]on (2.15) for $d_{t}=5$ . Deviations from the true solution can be observed in the middle part.

**Figure 11.**
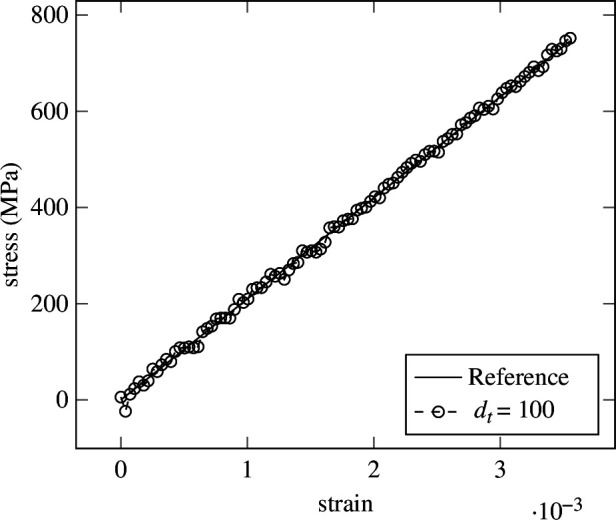
Elasticity—prediction in 100 strain (time) steps. Prediction of the LIF from equation (2.15) for $d_{t}=100$ . Fluctuations around the true solution can be observed.

It can be seen that the mean relative error is lowest for $d_{t}=5$ strain steps. For $d_{t}=2$ , the error is larger. This could be caused by a lack of a sufficient number of strain steps for the neuron dynamics to effectively be calculated. It can be understood as a failure due to too large strain steps in the explicit stepping scheme in equation (2.7). Clearly, the highest error can be observed for $d_{t}=100$ strain steps. In contrast, as depicted in figure 9, the error at the last strain step is lowest for $d_{t}=100$ strain steps. To illustrate the cause, the prediction of the network for two different samples, one for $d_{t}=5$ and one for $d_{t}=100$ strain steps are shown in figures 10 and 11, respectively. While good agreement on the endpoints is apparent, fluctuation during the rest of the strain steps causes the error to rise. Seemingly, the LIF has difficulties regressing a large number of strain steps. This could be caused by the lack of recurrent connections in the LIF formulation from equation (2.15), where history dependency is only weakly included in the form of the membrane potential. Therefore, recurrent LIFs will be introduced in §3.

*Remark*: The seemingly simple linear regression task provides a challenge for SNN, as effectively an ordinary differential equation has to be fitted to a linear function while relying on binary information transmission and inexact gradients.

## 3. Nonlinear regression using recurrent leaky integrate and fire

In order to counter the problems of vanishing information for a large number of strain (time) steps encountered in the preceding section, a recurrent SNN architecture is proposed (§3.1). Its performance is demonstrated by means of a numerical example in §3.2.

### 3.1. Recurrent leaky integrate and fire

The standard LIF is a feed-forward neuron, such that information is flowing unidirectionally in the form of spikes. By adding a feedback loop, a *recurrent LIF* (RLIF) can be formulated, which builds on the standard recurrent neural network (RNN) formulation. This enables the network to use relationships along several time steps for the prediction of the current time step. It was shown in Pascanu *et al*. [100] that recurrent loops can retain information for a relatively longer number of time steps when compared with their non-recurrent counterparts.

Here, the formulation of the hidden layer in equation (2.5) includes additional recurrent weights $V_{\eta }^{\left(l\right)}$ , such that


(3.1) $$h_{\eta ,t}^{\left(l\right)}=\phi^{\left(l\right)}\left(W_{\eta }^{\left(l\right)}h_{t}^{\left(l-1\right)}+V_{\eta }^{\left(l\right)}h_{t-1}^{\left(l-1\right)}\right).$$

In this RNN, the influence of the preceding time step is explicitly included by means of additional *recurrent weights* $V_{\eta }^{\left(l\right)}$ . The resulting set of trainable parameters reads


(3.2) $$\theta_{\text{RNN}}=\left\{W_{\eta }^{\left(l\right)},V_{\eta }^{\left(l\right)}\right\}.$$

The RNN formulation can be included in the LIF formulation from equation (2.7) to obtain an RLIF, such that


(3.3) $$\begin{array}{rl} U_{\eta ,t}^{\left(l\right)} & =\beta_{\eta }^{\left(l\right)}U_{\eta ,t-1}^{\left(l\right)}+W_{\eta }^{\left(l\right)}h_{t}^{\left(l-1\right)}\\ & \;+V_{\eta }^{\left(l\right)}h_{t-1}^{\left(l-1\right)}-\phi_{\text{spk},t-1}^{\left(l\right)}U_{\text{thr},\eta }^{\left(l\right)}, \end{array}$$

where $U_{\eta ,t}^{\left(l\right)}$ is again the membrane potential of the η th neural unit at time t , $U_{\text{thr},\eta }^{\left(l\right)}$ denotes the membrane threshold, $\beta_{\eta }^{\left(l\right)}$ is the membrane potential decay rate and $W_{\eta }^{\left(l\right)}h_{t}^{\left(l-1\right)}$ is the standard ANN weight multiplied with the preceding layer at the current time step. Additionally, $V_{\eta }^{\left(l\right)}$ denotes the recurrent weights from equation (3.1). This leads to the following set of trainable parameters


(3.4) $$\theta_{\text{RLIF}}=\left\{W_{\eta }^{\left(l\right)},V_{\eta }^{\left(l\right)},\beta_{\eta }^{\left(l\right)},U_{\text{thr},\eta }^{\left(l\right)}\right\}.$$

### 3.2. Numerical experiment: Ramberg–Osgood

The performance of the RLIF is investigated in nonlinear function regression. As a first test case, the nonlinear Ramberg–Osgood power law for modelling history-independent plasticity is considered, in which stress σ and strain ε are related via


(3.5) $$\varepsilon =\frac{\sigma }{E}+0.002{\left(\frac{\sigma }{\sigma_{Y}}\right)}^{n}.$$

Here, ε is the infinitesimal, one-dimensional elastic strain, σ denotes the one-dimensional Cauchy stress, E is Young’s modulus, n is a material constant describing the hardening behaviour of plastic deformation and $\sigma_{Y}$ is the yield strength of the material. Note that this plasticity model is only suited for a single loading direction and does not incorporate accumulation of plastic strain.

To this end, the general model described in §2.1 using RLIF defined in equation (3.3) is used. For a given strain history $\varepsilon_{t}$ , the stress response for different values of yield stress $\sigma_{Y}$ is predicted with the following parametric architecture


(3.6) $$\begin{array}{rl} S\left(\sigma_{Y}\right) & =h_{t}^{\text{pop}}\circ h_{t}^{\text{dec}}\circ h_{t}^{\text{RLIF}}\circ h_{t}^{\text{RLIF}}\circ h_{t}^{\text{RLIF}}\\ & \;\circ h_{t}^{\text{const}}\left(\sigma_{Y}\right)=\sigma_{t}. \end{array}$$

The training data consists of yield strength $\sigma_{Y}$ as input for fixed strains in the interval ε=[0,0.01] for $d_{t}=20$ strain steps. The yield strength is uniformly sampled in the interval $\sigma_{Y}=[100,500]$ MPa, and the stress output is calculated according to equation (3.5). The Young’s modulus is chosen as $E=2.1\times 10^{5}$ MPa and n=10 . Three datasets are generated, namely, training set, validation and test set with $n_{\text{train}}=n_{\text{val}}=n_{\text{test}}=1024$ samples, respectively. All three sets are standardized using the mean and standard deviation from the training set. The batch size is chosen as $n_{\text{batch}}=1024$ . The number of neurons $n_{u}$ is chosen as $n_{u}=128$ and is kept constant over all layers. The training is carried out for $5\times 10^{3}$ epochs. The model performing best on the validation set is chosen for subsequent evaluations. The mean relative error and the mean relative error of the last strain step with respect to the test set are reported.

**Figure 12.**
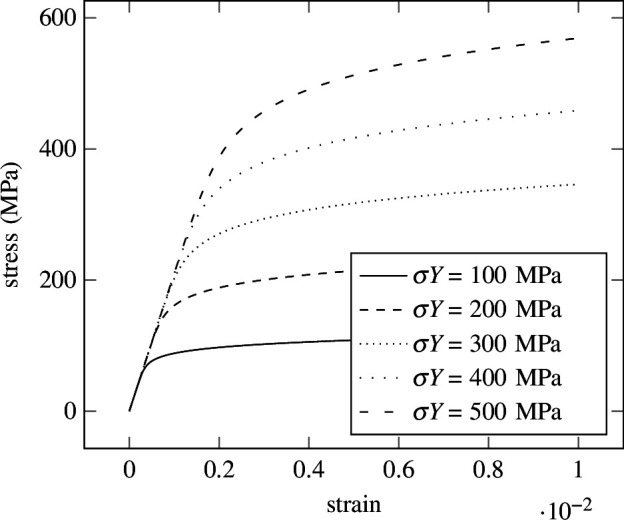
Ramberg–Osgood—reference solutions. Stress–strain curves of the Ramberg–Osgood material model for five different values of the yield stress $\sigma_{Y}$ obtained with Newton–Raphson algorithm.

**Figure 13.**
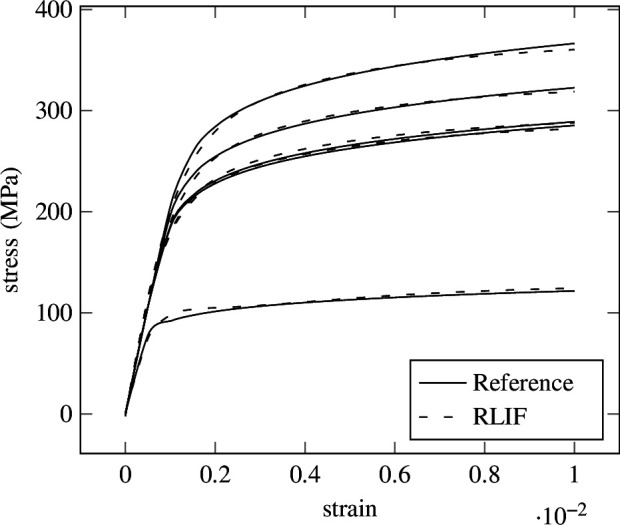
Ramberg–Osgood—RLIF prediction. Prediction of the RLIF from equation (3.6) for five different yield strength $\sigma_{Y}$ sampled from the test set for the nonlinear Ramberg–Osgood plasticity law.

**Figure 14.**
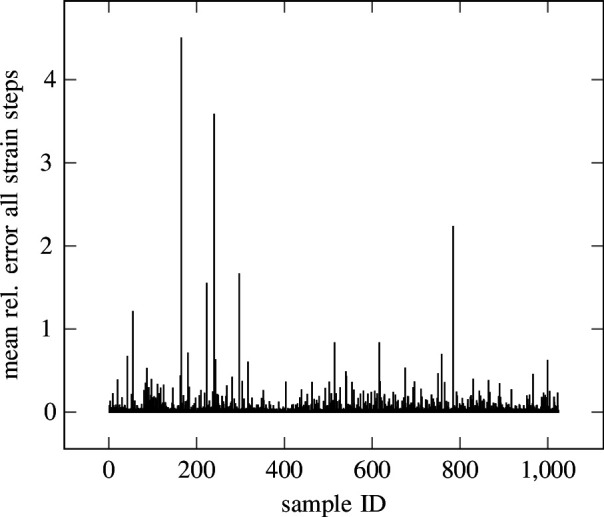
Ramberg–Osgood—RLIF test error. The mean relative error over all strain steps for 1024 samples from the test set for the numerical experiment is described in §3.2. It can be seen that some outliers have large error values, resulting in a mean error over all samples of $8.7934\times 10^{-2}$ . Most samples have a significantly lower error.

In figure 12, different stress–strain curves are depicted for different yield strength values, obtained by solving equation (3.5) for the stress σ with a classical Newton–Raphson method. The results of five different samples, randomly chosen from the $n_{test}=1024$ test samples, can be seen in figure 13. For the test set, a mean relative error for all strain steps of $8.7934\times 10^{-2}$ and a mean relative error for the last strain step of $8.0200\times 10^{-2}$ are obtained. The predictions on these five samples are more accurate than would be suspected from the mean relative error. The cause can be found in figure 14, where the mean relative error for all strain steps is plotted for every sample of the test set. It can be observed that a small number of samples has a much higher error than the rest, which impacts the error measure. This is caused by the purely data-driven nature of the experiment and can be tackled with approaches introduced in, for example, [101–103] .

Nevertheless, the RLIF is able to regress on the varying yield strength $\sigma_{Y}$ and can predict the resulting nonlinear stress–strain behaviour, as can be seen in the predictions (figure 13). Deviations can be observed around the yield point as well as the endpoints of the curves. To be able to take into account long-term history-dependent behaviour, the RLIF formulation will be expanded towards the incorporation of explicit long-term memory in the next section, where a more complex plasticity model is investigated.

## 4. History-dependent regression using spiking long short-term memory network

To extend the limited memory of the RNN in §3, a SLSTM (§4.1) is proposed. Previously, SLSTM hs been considered for classification problems [71]. Herein, a novel regression approach with a population decoding layer is discussed by means of a history-dependent plasticity model (§4.2).

### 4.1. Spiking long short-term memory network

A SLSTM is the spiking version of the standard LSTM [104], where the latter is defined as


(4.1) $$h_{\eta ,t}^{\left(l\right)}=o_{\eta ,t}^{\left(l\right)}⊙\phi_{\text{tanh}}\left(c_{\eta ,t}^{\left(l\right)}\right),$$

with


$$\begin{array}{rl} o_{\eta ,t}^{\left(l\right)} & =\phi_{\text{sigmoid}}\left(W_{o,\eta }^{\left(l\right)}h_{t}^{\left(l-1\right)}+V_{o,\eta }^{\left(l\right)}h_{t-1}^{\left(l-1\right)}\right),\\ c_{\eta ,t}^{\left(l\right)} & =f_{\eta ,t}^{\left(l\right)}⊙c_{\eta ,t-1}^{\left(l\right)}+i_{\eta ,t}^{\left(l\right)}⊙{\tilde{c}}_{\eta ,t}^{\left(l\right)},\\ f_{\eta ,t}^{\left(l\right)} & =\phi_{\text{sigmoid}}\left(W_{f,\eta }^{\left(l\right)}h_{t}^{\left(l-1\right)}+V_{f,\eta }^{\left(l\right)}h_{t-1}^{\left(l-1\right)}\right),\\ i_{\eta ,t}^{\left(l\right)} & =\phi_{\text{sigmoid}}\left(W_{i,\eta }^{\left(l\right)}h_{t}^{\left(l-1\right)}+V_{i,\eta }^{\left(l\right)}h_{t-1}^{\left(l-1\right)}\right),\\ {\tilde{c}}_{\eta ,t}^{\left(l\right)} & =\phi_{\text{tanh}}\left(W_{c,\eta }^{\left(l\right)}h_{t}^{\left(l-1\right)}+V_{c,\eta }^{\left(l\right)}h_{t-1}^{\left(l-1\right)}\right), \end{array}$$

where $f_{t}$ denotes the *forget gate* with *sigmoid activation* $\phi_{\text{sigmoid}}$ or *tangent hyperbolicus activation* $\phi_{\text{tanh}}$ and corresponding weights $W_{f},V_{f}$ with absorbed biases. The same nomenclature holds for the *input gate* $i_{t}$ , the *output gate* $o_{t}$ , the *cell input* ${\tilde{c}}_{t}$ and the *cell state* $c_{t}$ with their respective activations and weights. The new cell state $c_{t}$ and the output of the LSTM $h_{t}$ are formed using the Hadamard or point-wise product ⊙ . The parameters of the LSTM are its weights, such that


(4.2) $$\begin{array}{rl} \theta_{\text{LSTM}}=\{ & W_{f,\eta }^{\left(l\right)},W_{i,\eta }^{\left(l\right)},W_{o,\eta }^{\left(l\right)},W_{c,\eta }^{\left(l\right)},\\ & V_{f,\eta }^{\left(l\right)},V_{i,\eta }^{\left(l\right)},V_{o,\eta }^{\left(l\right)},V_{c,\eta }^{\left(l\right)}.\}. \end{array}$$

For detailed derivations and explanations of standard LSTM, see, for example, [85,86]. The SLSTM can be obtained from the LSTM by using spike activations within the LSTM formulation from equation (4.1) , such that


(4.3) $$h_{\eta ,t}^{\left(l\right)}=o_{\eta ,t}^{\left(l\right)}⊙\phi_{\text{tanh}}\left(c_{\eta ,t}^{\left(l\right)}\right)-\phi_{\text{spk},t-1}^{\left(l\right)}U_{\text{thr},\eta }^{\left(l\right)},$$

where the output $h_{\eta ,t}^{\left(l\right)}$ is used to determine if a spike is produced


(4.4) $$\begin{array}{r} \phi_{\text{spk},t}^{\left(l\right)}=\left\{ \begin{array}{l} 1,\;h_{\eta ,t}^{\left(l\right)}\geq U_{\text{thr},\eta }^{\left(l\right)}\\ 0,\;h_{\eta ,t}^{\left(l\right)}<U_{\text{thr},\eta }^{\left(l\right)}. \end{array}\right. \end{array}$$

In other words, the output of $h_{\eta ,t}^{\left(l\right)}$ can be interpreted as the membrane potential of the SLSTM, such that $h_{\eta ,t}^{\left(l\right)}=U_{\eta ,t}^{\left(l\right)}$ . A decay parameter β is not used in this formulation. Rather than using decay to remove information from the cell state $c_{\eta ,t}^{\left(l\right)}$ , this is achieved by carefully regulated gates. The corresponding optimization parameters of the SLSTM are


(4.5) $$\begin{array}{rl} \theta_{\text{SLSTM}}=\{ & W_{f,\eta }^{\left(l\right)},W_{i,\eta }^{\left(l\right)},W_{o,\eta }^{\left(l\right)},W_{c,\eta }^{\left(l\right)},\\ & V_{f,\eta }^{\left(l\right)},V_{i,\eta }^{\left(l\right)},V_{o,\eta }^{\left(l\right)},V_{c,\eta }^{\left(l\right)},U_{\text{thr},\eta }^{\left(l\right)}\}. \end{array}$$

Basically, the cell state $c_{\eta ,t}^{\left(l\right)}$ acts as long-term memory, just like in the standard LSTM formulation. The communication between layers is handled via spike trains that depend on the membrane potential $h_{\eta ,t}^{\left(l\right)}=U_{\eta ,t}^{\left(l\right)}$ in equation(4.3) and the activation function $\phi_{\text{spk},t}^{\left(l\right)}$ from equation (4.4) .

### 4.2. Numerical experiment: Isotropic hardening using spiking long short-term memory network

The following numerical experiments aim to investigate the performance of the proposed SLSTM on nonlinear, history-dependent problems. Therefore, a one-dimensional plasticity model with isotropic hardening is investigated. Following Simo and Hughes [105], the model is defined by


(4.6) $$\begin{array}{rl} & 1.\;\varepsilon =\varepsilon_{\text{el}}+\varepsilon_{\text{pl}},\\ & 2.\;\sigma =E\left(\varepsilon -\varepsilon_{\text{pl}}\right),\\ & 3.\;{\dot{\varepsilon }}_{\text{pl}}=\gamma \mathrm{sign}\left(\sigma \right),\;\dot{\alpha }=\gamma ,\\ & 4.\;f\left(\sigma ,\alpha \right)=|\sigma |-\left(\sigma_{Y}+K\alpha \right)\leq 0,\\ & 5.\;\gamma \geq 0,\;f\left(\sigma ,\alpha \right)\leq 0,\;\gamma f\left(\sigma ,\alpha \right)=0,\\ & 6.\;\gamma \dot{f}\left(\sigma ,\alpha \right)=0,\;\text{if}\;f\left(\sigma ,\alpha \right)=0, \end{array}$$

where

1. is the additive elastoplastic split of the small-strain tensor ε into a purely elastic part $\varepsilon_{\text{el}}$ and a purely plastic part $\varepsilon_{\text{el}}$ ;
2. denotes the elastic stress–strain relationship for the Cauchy stress tensor σ and elastic modulus E ;
3. describes the flow rule and isotropic hardening law with consistency parameter γ and equivalent plastic strain α ;
4. gives the yield condition f(σ,α) with hardening modulus K ;
5. denotes the Kuhn–Tucker complementarity conditions; and
6. describes the consistency condition.

**Figure 15.**
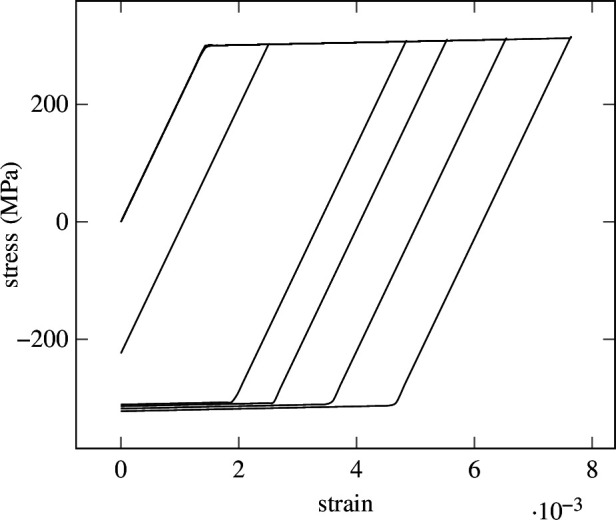
Isotropic hardening—reference solutions. Five stress–strain curves sampled from the isotropic hardening material model for different maximum strains obtained from equation (4.6).

In figure 15, different stress–strain paths are shown for varying strains. Especially long-time dependencies are of interest. To this end, the predictive capabilities of the SNN are investigated for inference over $d_{t}=100$ strain steps, where the elastoplastic model is evaluated using a classical explicit return-mapping algorithm, see Simo and Hughes [105].

The training data consists of strain as input, uniformly sampled in the interval ε=[0,0.01] , and stress as output calculated according to equation (4.6). The yield stress is chosen as $\sigma_{Y}=300$ MPa, the elastic modulus $E=2.1\times 10^{5}$ MPa and the hardening modulus as $2.1\times 10^{4}$ MPa. Three datasets are generated, namely, a training set with $n_{\text{train}}=10\;240$ samples and a validation set and test set with $n_{\text{val}}=n_{\text{test}}=1024$ samples. All three sets are standardized using the mean and standard deviation from the training set. The batch size is chosen as $n_{\text{batch}}=1024$ . The training is carried out for 500 epochs. The model performing best on the validation set is chosen for subsequent evaluations. The mean relative error accumulated over all time steps and the mean relative error of the last strain step with respect to the test set are reported.

#### 4.2.1. Influence of hyperparameter

The first study investigates the prediction accuracy as a function of (i) the number of output neurons, which participate in the population regression outlined in §2.1 and (ii) different capacities of the SLSTM in the sense of layer width. To this end, the SLSTM defined in equation (4.3) is used, resulting in the following architecture:


(4.7) $$\begin{array}{rl} \;S_{\text{SLSTM}}\left(\varepsilon_{t}\right) & =h_{t}^{\text{pop}}\circ h_{t}^{\text{dec}}\circ h_{t}^{\text{SLSTM}}\circ h_{t}^{\text{SLSTM}}\circ h_{t}^{\text{SLSTM}}\\ & \;\circ h_{t}^{\text{const}}\left(\varepsilon_{t}\right)=\sigma_{t}. \end{array}$$

**Figure 16.**
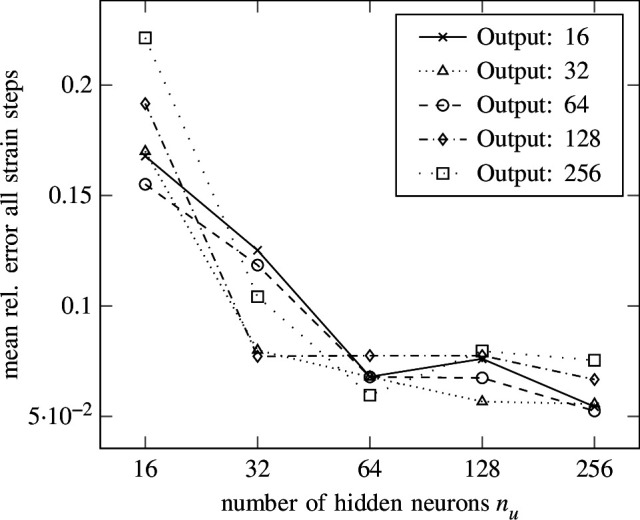
Isotropic hardening—error versus width. The mean relative error of the last strain step versus the number of hidden neurons per layer is shown for different numbers of output neurons in the isotropic hardening experiment from §4 using the SLSTM from equation (4.7).

**Figure 17.**
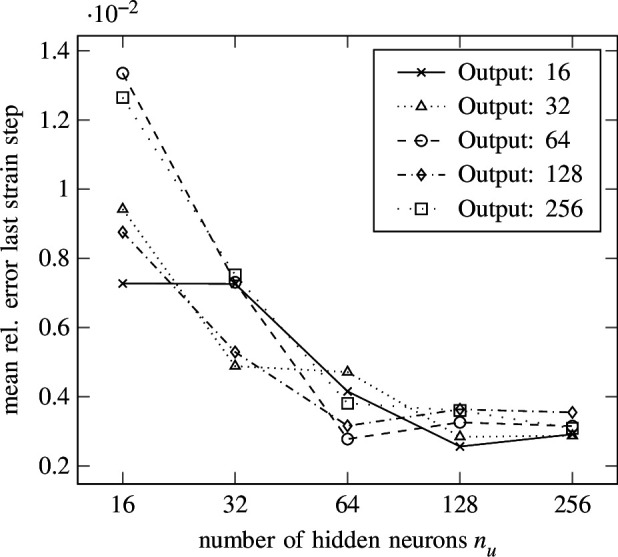
Isotropic hardening—error versus width. The mean relative error of all strain steps versus the number of hidden neurons per layer is shown for different numbers of output neurons in the isotropic hardening experiment from §4 using the SLSTM from equation (4.7) .

Multiple simulations with output neurons and hidden layers drawn from the grid $n_{u}\times n_{o}=[16,32,64,128,256]\times [16,32,64,128,256]$ are carried out. The resulting mean relative error for all strain steps with respect to the test set is shown in figure 16, whereas the resulting mean relative error of the last strain step with respect to the test set is depicted in figure 17. A clear convergence behaviour can be observed for the number of hidden neurons $n_{u}$ , where larger numbers of neurons lead to lower errors. For the number of output neurons $n_{o}$ , a tendency can be observed upon convergence with respect to $n_{u}$ . For the largest number of hidden neurons $n_{u}=256$ , the mean relative error over all strain steps and the mean relative error of the last strain step get larger for $n_{o}=[128,256]$ output neurons, whereas for $n_{o}=[16,32,64]$ the errors are almost the same. The lowest mean relative error for all strain steps is $5.2445\times 10^{-2}$ for $n_{u}=256$ hidden neurons per layer and $n_{o}=64$ output neurons. The lowest mean relative error for the last strain steps is $2.8729\times 10^{-3}n_{u}=256$ hidden neurons per layer and $n_{o}=32$ output neurons. Again, the seemingly high errors are caused by outliers polluting the average, as also observed in §3.2. The same counter-measures discussed in §3.2 can be applied to prohibit outliers, for example, by enforcing thermodynamic consistency.

#### 4.2.2. Energy and memory efficiency

**Figure 18.**
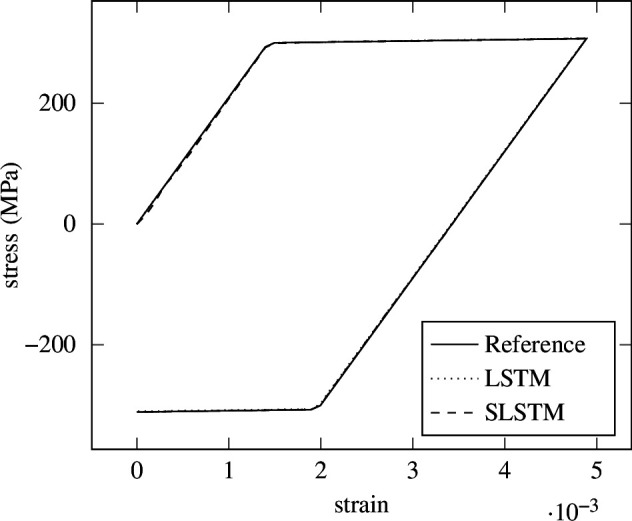
Isotropic hardening—LSTM versus SLSTM. Prediction of a single load path using the return-mapping algorithm as a reference, the standard LSTM and the spiking LSTM formulation.

For the second experiment, the SLSTM using $n_{o}=64$ output neurons and $n_{u}=256$ hidden neurons per layer are compared with a standard LSTM with an equal number of optimization parameters. The aim of this study is the comparison of the prediction accuracy, but also the difference in memory and energy consumption on neuromorphic hardware. For both ANN variants to be comparable, the same topology is chosen for the LSTM as for the SLSTM, such that


(4.8) $$\begin{array}{rl} N_{\text{LSTM}}\left(\varepsilon_{t}\right) & =h_{t}^{\text{dense}}\circ h_{t}^{\text{dense}}\circ h_{t}^{\text{LSTM}}\circ h_{t}^{\text{LSTM}}\circ h_{t}^{\text{LSTM}}\\ & \;\circ h_{t}^{\text{const}}\left(\varepsilon_{t}\right)=\sigma_{t}, \end{array}$$

where the last two layers are replaced by densely connected conventional feed-forward neural networks. Again, the training was carried out for $5\times 10^{3}$ epochs and the same datasets from the previous experiments are used. The standard LSTM from equation (4.8) reached a mean relative error of $4.8611\times 10^{-2}$ over all strain steps and a mean relative error of $4.7569\times 10^{-3}$ for the last strain step. The SLSTM from equation (4.7) reached a mean relative error of $9.3832\times 10^{-2}$ over all strain steps and a mean relative error of $4.0497\times 10^{-3}$ for the last strain step. The resulting prediction for one strain path is illustrated in figure 18. Clearly, both networks are able to accurately predict the history-dependent, nonlinear stress–strain behaviour.

Some deviations from the SLSTM can be seen at the beginning of the curve. The dynamics of the spiking formulation result in a higher mean relative error over all strain steps with respect to the LSTM. However, the endpoint has a better fit than the LSTM. This is seen in the lower error at the last strain step.

To assess the potential of interfacing our model in embedded, resource-constrained sensors in the wild, we performed a series of power profiling experiments for our SNNs (both using LIF neurons and SLSTMs) when processed on the Loihi neuromorphic chip [54]. These results are compared against their non-spiking equivalents on an NVIDIA V100 GPU. Data were extracted using the energy profiler in KerasSpiking v. 0.3.0. The emulation tool takes all non-zero activations, accumulates the operation count of each activation (i.e. the fan-out of the activated neuron) and scales this by the estimated energy cost per operation. Specifically, this operation is a ‘read-and-accumulate’, where the presence of a spike requires all connected synapses to be ‘read’ from memory and accumulated with other terms. Other literature refers to this as ‘SynOps’ or ‘Synaptic Operations’ [106–110]. This is a coarse, but reasonable, approximation for small-scale models as neuromorphic hardware skips processing non-zero activations. For large-scale models that require inter-chip data communication, the energy cost of overhead is not accounted for in such a model. For our purposes, with lightweight, low-power models, this is an acceptable approximation. It is also the de facto metric for measuring energy efficiency *in silico* from the NeuroBench [110] initiative, which aims to find representative benchmarking of neuromorphic models.

The first difference in energy usage is that the spiking implementation is measured in an ‘event-based’ manner, where processing only occurs when a neuron emits a spike. In contrast, a non-spiking network processed on a GPU continuously computes with all activations. The second difference is that SNNs require multiple strain steps of a forward pass, whereas their non-spiking counterparts do not (unless the input to the network varies over time). Note that the cost of overhead did not need to be accounted for (i.e. transferring data between devices) because all models fit on a single device.

Each network has been broken up into its constituent layers to measure how much they contribute to energy usage on each device. The total energy consumption per forward pass of the non-spiking network on the V100 is 512 nJ, whereas the equivalent SNN is 4.25 nJ. This represents a 120× reduction in energy consumption. The non-spiking LSTM network consumed 5.7 µJ while the proposed SLSTM architecture required 24 nJ, a 238× reduction. Detailed results are summarized in table 1.

**Table 1.** Comparison between spiking and non-spiking forward-pass energy consumption and memory usage.

architecture	energy		architecture	energy	
dense	Loihi (nJ)	GPU (nJ)	LSTM	Loihi (nJ)	GPU (nJ)
FC1	6.9e-2	0.61	LSTM1	0.28	2.5
FC2	1.3	160	LSTM2	11	2.5e3
FC3	1.3	160	LSTM3	10	2.5e3
FC4	1.2	160	FC1	2.6	630
FC5	0.3	39	FC2	0.21	39
total energy	4.25	512	total energy	24	5.7e3
reduction	×120		reduction factor	×238	
synaptic memory	0.86 MB		synaptic memory	9.5 MB	

## 5. Conclusion and outlook

In the present study, a framework for regression using SNNs was proposed based on a membrane potential spiking decoder and a population voting layer. Several numerical examples using different spiking neural architectures investigated the performance of the introduced topology towards linear, nonlinear and history-dependent regression problems.

First, a simple feed-forward SNN, the LIF, was derived from the classical densely connected feed-forward ANN. It was shown that the SNN can be seen as a special kind of activation function, which produces binary outputs, so-called spikes. These spikes are used to propagate information through a possibly deep SNN. The spikes occur due to the dynamic behaviour of the membrane potential inside the neuron, which rises when spikes appear at the input and decays over time if no spikes appear. If a certain threshold value is reached, the membrane potential is reset and the neuron emits a spike itself. This formulation introduces more hyperparameters, which fortunately can be learned during training. The spikes introduce sparsity in the network, which can be effectively exploited by neuromorphic hardware to improve latency, power and memory efficiency. The non-differentiability of the binary spikes is circumvented by surrogate gradients during backpropagation.

Next, a network topology was proposed, which decodes binary spikes into real numbers, which is essential for all kinds of regression problems. A decoding layer takes the membrane potentials of all neurons in the last spiking layer and propagates them to a population voting layer, which provides its mean potential resulting in a real number. The proposed topology can be used for arbitrary temporal input and output dimensions. A simple experiment on a linear elastic material model using LIFs showed that the proposed topology is able to regress the problem. It was shown that errors are introduced for a large number of strain steps. This problem was overcome by introducing RLIF, which extends the LIF by recurrent feedback loops. An experiment using a nonlinear Ramberg–Osgood plasticity model showed that the proposed topology using RLIF is able to regress varying yield limits accurately. The final extension was concerned with the introduction of explicit long-term memories inspired by the classical LSTM formulation, resulting in a spiking LSTM. The performance of this SLSTM was investigated on a history-dependent isotropic hardening model, where different load paths were accurately regressed. During prediction, the SLSTM was able to generalize even better than the LSTM for the final load step. Furthermore, the convergence of the proposed method was shown. Note that an extension towards two- and three-dimensional mechanical problems is equally possible. This will involve learning a functional relationship for each stress component, which can be either achieved by a multi-input/output architecture or by using individual networks for each stress component. This treatment is analogous to second-generation ANN, see for instance [111], where stress data has been decoupled using Proper orthogonal decomposition (POD).

Power profiling and memory analysis were conducted on the LIF and SLSTM networks to compare efficiency on neuromorphic hardware as against a GPU. The Loihi neuromorphic processor was able to achieve a 120× reduction in energy consumption when processing the dense LIF network, and the SLSTM offered a 238× reduction in energy during inference.

The range of possible future application scenarios enabled by regression with SNN are manifold. For instance, today’s sensing systems cannot capture all quantities that are relevant for structural health monitoring. In the context of mechanics, displacement and strain are quite easy to assess, but the mechanical stress, which reflects the actual response of structures and materials to deformation, remains a so-called *hidden quantity*. Physics-informed machine learning offers the potential to reconstruct *hidden quantities* from data by leveraging information from physical models, given in the form of partial differential equations. It is expected that the developments in the field of neuromorphic hardware will foster the development of a new generation of embedded systems, which will ultimately enable control of structures and processes based on partial differential equations.

## Data Availability

The code is available at: https://github.com/ahenkes1/HENKES_SNN and Zenodo [112].
